# Emotional Responses to Bed Bug Encounters: Effects of Sex, Proximity, and Educational Intervention on Fear and Disgust Perceptions

**DOI:** 10.3390/insects16080759

**Published:** 2025-07-24

**Authors:** Corraine A. McNeill, Rose H. Danek

**Affiliations:** 1 Department of Natural and Mathematical Sciences, Morningside University, 1501 Morningside Avenue, Sioux City, IA 51106, USA; 2 Department of Behavioral and Social Sciences, Embry-Riddle Aeronautical University, 3700 Willow Creek Road, Prescott, AZ 86301, USA; danekr1@erau.edu

**Keywords:** bed bug, *Cimex lectularius*, emotion, anger, disgust, fear

## Abstract

This study investigated individuals’ emotional responses to bed bugs and how these were influenced by sex, proximity, and educational intervention. Using a pre-post experimental design, participants (*n* = 157) completed emotional assessments before and after viewing an educational video about bed bugs. Contrary to our initial hypothesis that only fear and disgust would be observed, participants also exhibited high levels of anxiety and anger. Following the educational intervention, disgust, fear, and anger toward bed bugs increased significantly. Participants experienced greater disgust and fear when imagining encounters with bed bugs in closer proximity, with home infestations giving stronger responses than workplace scenarios. The educational video reduced disgust toward bed bugs in the home but increased fear of them in public spaces, potentially promoting vigilance that could limit bed bug spread. Females reported higher levels of disgust and fear than males across all proximity conditions, supporting evolutionary theories regarding sex-specific disgust sensitivity. The educational video successfully increased participants’ knowledge about bed bugs while simultaneously shifting emotional responses from contamination-based disgust to threat-specific fear. These findings suggest that educational interventions can effectively modify emotional responses to bed bugs, potentially leading to more rational management behaviors by transforming vague anxiety into actionable awareness of specific threats.

## Simple Summary

Bed bugs trigger strong emotional reactions in people, including fear, disgust, anxiety, and anger. This study examined how these emotions vary by sex, proximity to infestation, and educational intervention. Researchers measured emotional responses in 157 participants before and after they watched an educational video about bed bugs. Contrary to expectations, people experienced not just fear and disgust, but also significant anxiety and anger when thinking about bed bugs. Surprisingly, the video increased feelings of fear, disgust, and anger toward bed bugs, though it also boosted knowledge about the pest. People felt stronger negative emotions when imagining bed bugs in their homes versus workplaces. Women reported stronger feelings of disgust and fear than men in all scenarios, supporting evolutionary theories about sex differences in disgust sensitivity. This educational video, which reduced people’s disgust about bed bug home infestations, but increased their fear of bed bugs in public spaces, could promote vigilance that helps prevent bed bug spread. Overall, the study shows that targeted education can shift how people think and feel about bed bugs, turning general anxiety into focused awareness. This approach may help communities respond to bed bug infestations more effectively and manage them with clearer, more rational actions.

## 1. Introduction

Bed bugs (*Cimex lectularius*) are parasitic insects that feed on human and animal blood, causing significant physical and psychological consequences [1,2]. While the COVID-19 pandemic temporarily reduced bed bug sightings by approximately 20% due to travel restrictions [3], their prevalence surged following the removal of these restrictions, leading to what became known in Europe as the “Great Big Bed Bug Outbreak” [4,5].

These pests represent a substantial public health concern [6], with infestations resulting in physical symptoms, secondary infections, allergies, and various health complications [7]. The economic burden is considerable, particularly affecting underserved communities [8] and homeless shelters [9]. Management often requires multiple treatments and disposal of belongings [10].

The psychological consequences of bed bug infestations are profound, including anxiety, nightmares, phobias, hypervigilance, insomnia, and diminished self-esteem [11]. These effects can lead to personal dysfunction and avoidance behaviors [12], with infestations acting as significant stressors on mental health [13]. In extreme cases, the psychological distress has been associated with suicide [12,14].

Bed bug infestations can trigger such intense fear and aversion that people resort to extreme elimination methods, sometimes with catastrophic consequences, including apartment complex fires [15,16], setting themselves on fire [17] and rental vehicle explosions in public parking areas [18]. Many of these behaviors stem from misconceptions about bed bugs and safe and effective control strategies.

One potential motivation for these extreme behaviors could be emotional reactions to bed bug infestations. Research on emotional perceptions towards insects generally reveal aversion, fear, or indifference [19,20,21]. While some insects elicit positive emotions (e.g., butterflies, bees), others trigger negative responses (e.g., flies, mosquitoes) [21,22]. Notably, insects are more tolerated outdoors than in domestic environments [21], with disgust being a common reaction to many invertebrates [23].

Emotions function as adaptive, motivating forces guiding behavior in important situations [24]. Discrete emotion theory identifies core emotions (anger, disgust, fear, sadness, joy/happiness) that are fundamentally distinguishable through expression, physiology, and stimuli [25,26]. These emotions can be categorized by valence (pleasant/unpleasant), arousal level, and motivational direction (approach/avoidance) [27,28,29,30]. While anger, disgust, and fear are all negatively valanced with high arousal, anger typically motivates approach behaviors, whereas disgust and fear prompt avoidance [26].

The emotional impact of bed bug infestations can trigger extreme behavioral responses, yet research specifically addressing these psychological reactions remains surprisingly scarce. This study addresses this critical knowledge gap in the literature by examining people’s emotional responses to bed bugs. Despite the widespread impact of bed bug infestations, empirical studies on their psychological effects are limited, likely due to the stigma associated with these pests [31,32]. While anecdotal evidence suggests bed bugs evoke stronger negative emotions than other urban pests, often leading to extreme behaviors [32], systematic research is needed. Understanding the primary emotional responses of individuals towards bed bugs is crucial as this directly influences their perception, reaction, and subsequent behaviors toward these urban pests. To address these research needs, this study had three interconnected objectives:

(1) Examine the emotional responses to bed bugs by:
  (a) establishing baseline emotional perceptions.
  (b) tracking changes in emotional intensity following the viewing of educational video content.
(2) Analyze variations in emotional responses (particularly fear and disgust) based on:
  (a) Sex differences.
  (b) Proximity by environmental contexts (e.g., inside versus outside and home versus public settings).
  (c) Knowledge of bed bugs.
(3) Evaluate the effectiveness of a brief, self-guided educational video in improving public knowledge about bed bug management and control strategies.

## 2. Materials and Methods

This study was a comprehensive attempt to gain insight into the emotions, perceptions, and potential behaviors associated with bed bugs. A series of survey questions and a short video were used to examine these factors.

### 2.1. Participants

Participants were recruited from a small private university in the southwestern United States. Prior to recruiting participants, the project was approved by the Institutional Review Board (#23-039) and all ethical guidelines were followed. All the participants provided informed consent but were naïve as to the study’s design and purpose. Participants completed the entire study in a single session lasting approximately 30–45 min depending on the speed of the participant. Of the 174 initial participants, 17 were excluded from analysis: 8 did not complete both surveys, 1 did not watch the informational video, 3 provided incorrect ID numbers that prevented matching their data, and 5 did report their sex. Five participants were removed due to not marking male or female as sex. Of the remaining 157 participants, 98 (62.4%) were male and 59 (37.6%) were female. Participants ranged in age from 18 to 32 with a mean age of 19.48 (*SD* = 2.23). Twenty-seven (17.2%) participants reported being multiracial, 21 (13.4%) were Asian, 4 (2.5%) were Black or African American, 8 (5.1%) were Hispanic or Latino, 1 (0.6%) was Middle Eastern or North African, 1 (0.6%) was a Native Hawaiian, 94 (59.9%) were White, and 1 (0.6%) preferred not to answer. The majority of the participants had at least some college, while three (1.9%) participants had an associate’s degree, and two (1.3%) participants had completed a bachelor’s degree. Of the participants surveyed, the majority of them (135 participants; 85.9%) had never had direct experience with bed bugs. The remaining 22 participants (14.1%) had at least some direct experience with bed bugs (e.g., in their home, at work or school, in temporary lodgings).

### 2.2. Experimental Procedure

The materials consisted of a pretest survey which included demographic information (i.e., age, sex, race/ethnicity, education level, and previous experience with bed bugs). The next portion of the survey was a scale designed by the researchers to measure disgust and fear if an encounter with a bed bug or a butterfly (another common urban insect; used as a “control”) occurred inside or outside of your home or work (see Appendix A). Also included was a modified version of the Discrete Emotions Questionnaire (DEQ) [26] used to look at the emotions of fear, anger, anxiety, happiness, disgust, and relaxation when thinking about bed bugs or butterflies along with two free responses questions asking participants to describe the feelings evoked by bed bugs or butterflies. The DEQ scale ranges from “1” meaning “not at all” to “7” meaning “an extreme amount.” Finally, participants took a 17-item knowledge test on bed bugs which was modified from a previous scale by one of the authors (see Appendix B; [33]).

Participants then watched a 10 min pre-recorded narrated PowerPoint presentation about bed bugs which was generated and narrated by one of the authors. This PowerPoint and narration were in the form of a video that participants watched. For the posttest, which was given after the video, participants answered all the same surveys that were in the pretest, except demographics were not recollected.

All surveys and the video were administered via the participants’ own phones. Each participant was asked to bring their phone and a pair of headphones/earbuds to the experiment. Upon arrival at the lab, participants received an ID number on an index card upon arrival and then were given a self-guided packet containing QR codes leading to the pretest survey, video, and posttest survey. The research assistants explained that the instructions for the study were in the packet and noted that participants would start the first survey using the QR code on the first page, and then the survey would tell them to go to the next page, watch the video, and then go on to the last survey using the QR code on the final page. Participants were given the chance to ask any clarification questions. Once all questions were answered, if any, participants were given the materials (QR packet and ID card) and allowed to begin.

Once the participants were settled, Research Assistants faced away from the participants so that they were not directly watching the participants take the surveys. When participants were finished, they turned in the QR packets and ID index cards, were given a copy of the debriefing, credit for their participation in the SONA system (Sona Systems, Ltd., Tallinn, Estonia), and thanked.

## 3. Results

### 3.1. Emotional Perceptions of Bed Bugs: Changes in Fear and Disgust Following Educational Intervention

**Figure 1:**
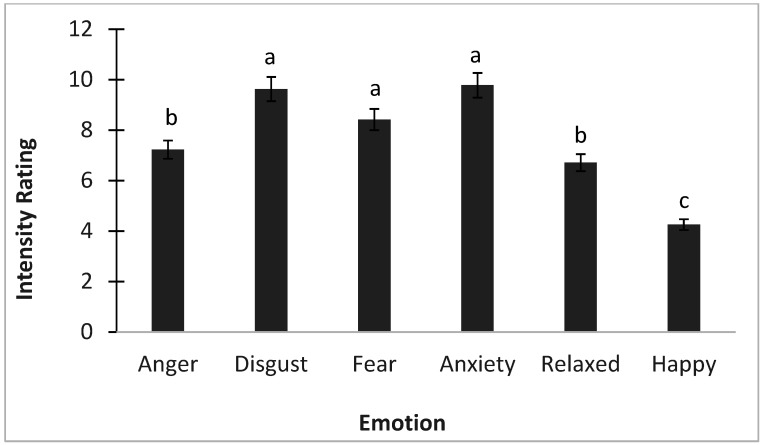
Emotion responses to bed bugs (mean ± SE) based on the Discrete Emotions Questionnaire. Different letters indicate significantly different means.

To understand the emotional perceptions of bed bugs, participants filled out a modified version of the Discrete Emotions Questionnaire [26]. To keep the study to a reasonable time, and to measure emotions that were hypothesized to directly influence pest management behaviors and decision-making, desire and sadness were not tested. The three top reported emotions that individuals exhibited regarding bed bugs were disgust, anxiety, and fear (Figure 1).

**Figure 2:**
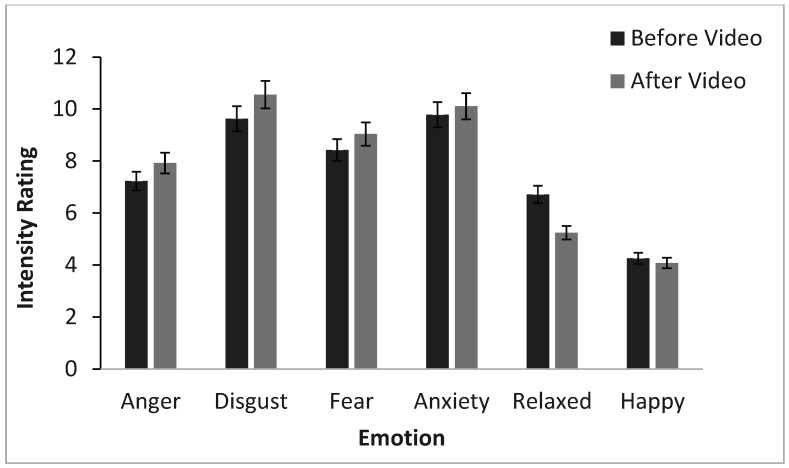
Pre and posttest emotional responses (mean ± SE) to bed bugs based on the Discrete Emotions Questionnaire.

While the top three emotions reported about bed bugs remained disgust, anxiety, and fear after the video was shown, four of the six emotions tested on the DEQ showed statistically significant differences in intensity. These differences were observed between pre-video intervention and post-video intervention measurements. In the posttest, there was no statistically significant change in anxiety or happiness levels, but anger (*t*(151) = −4.55, *p* < 0.001), disgust (*t*(144) = −4.49, *p* < 0.001), and fear (*t*(151) = −2.88, *p* = 0.005) significantly increased after watching the bed bug video, while relaxation decreased after watching the video (*t*(151) = 7.28, *p* < 0.001) (Figure 2).

Participants rated their anger, on average, near “somewhat” (before = 7.21; after = 7.95), their disgust near “somewhat” (before = 9.50; after = 10.47), their fear as a bit above “somewhat” (before = 8.35; after = 9.01), and their relaxation from “slightly” to “not at all” (before = 6.76; after = 5.26). Anxiety and happiness had no statistically significant change before and after the video, but anxiety was a bit above “somewhat” (9.75) and happiness was almost “not at all” (4.27).

In stark contrast to what was observed for bed bugs, the reactions to butterflies were very different. The top self-reported emotions evoked by butterflies and captured by the DEQ were relaxation and happiness by far. Relaxation was rated between “moderate” and “quite a bit” (14.16), while happiness was around “moderate” levels (12.97). All the other emotions (anger, disgust, anxiety, fear) were reported around levels of “not at all” (ranging from 4.10 to 4.26). After the video, relaxation decreased slightly for butterflies *t*(153) = 2.37, *p* = 0.019, becoming slightly closer to “moderate” (13.70), which could be expected given butterflies were not mentioned in the study outside of the surveys, but “bugs” were heightened in salience. These data indicate that bed bugs are certainly viewed in a more negative manner than at least one other common urban insect. Previous research has shown that there are differences in perception of different insects but have not looked specifically at bed bugs [19,20,21,22].

### 3.2. Sex, Proximity, and Prior Knowledge: Influences on Emotional Responses to Bed Bug Encounters

Before collecting DEQ data, it was hypothesized that fear and disgust would be two emotions evoked in response to bed bugs. These emotions were further tested with a set of questions asking participants to rate their level of fear or disgust when thinking about encountering bed bugs in places varying in proximity to themselves (inside vs. outside) and places personal to the participant (home vs. work).

Prior to the video, participants rated their disgust and fear of seeing bed bugs at varying levels of proximity and personal space on a scale from not at all (1) to an extreme amount (5). Participants had the highest disgust rating (quite a bit) for inside the home (*M* = 3.81), then (“moderate”) inside their work (*M* = 3.37), then between “somewhat” and “moderate” for outside the home (*M* = 2.56), and finally the least (“somewhat”) for outside of work (*M* = 2.41). A three-way mixed ANOVA was conducted to examine the effects of time (before or after video); proximity (inside/outside/home/work), and sex (male or female) on disgust ratings. There was a statistically significant main effect of proximity using the Greenhouse-Geisser correction *F*(2.15, 307.70) = 239.40, *p* < 0.001. LSD post hoc tests revealed that each of these levels was statistically significantly different from each other.

Another three-way ANOVA was conducted to determine the effects of time, proximity, and sex on fear ratings. In terms of proximity, the same was true for fear as was true for disgust, inside the home (*M* = 3.13) was the most fear evoking followed by inside work (*M* = 2.03), outside the home (*M* = 2.68), and finally outside of work (*M* = 1.88). All differences were statistically significant with the Greenhouse-Geisser correction *F*(2.08, 276.65) = 171.71, *p* < 0.001. Showing a clear trend for proximity and personal space being important.

For fear, our analysis of bed bug emotional responses revealed significant effects across proximity (*F*(2.08, 276.65) = 171.71, *p* < 0.001), time (*F*(1, 133) = 9.12, *p* = 0.003), and sex (*F*(1, 133) = 997.12, *p* < 0.001), the three-way interaction was significant (*F*(2.67, 355.37) = 4.86, *p* = 0.004), and the time-proximity (*F*(2.67, 355.37) = 9.68, *p* < 0.001) and proximity-sex (*F*(2.08, 276.65) = 4.10, *p* = 0.007) interactions were also statistically significant, the time-sex interaction was not statistically significant (*F*(1, 133) = 0.10, *p* = 0.747).

**Figure 3:**
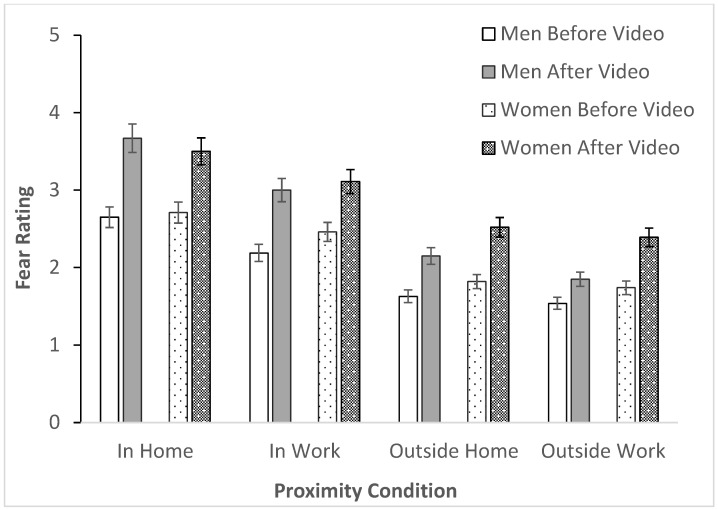
Three-way interaction (sex, time, proximity) to fear with (mean ± SE).

Fear responses showed clear patterns: females reported significantly higher fear than males; all participants feared bed bugs most in home environments compared to work or outside locations; and fear increased after video exposure for both sexes (Figure 3).

The proximity-sex interaction maintained this pattern, with both sexes showing highest fear for home infestations (Figure 3). The time-proximity interaction revealed increased fear post-video across workplace, outside home, and outside work contexts (Figure 3).

**Figure 4:**
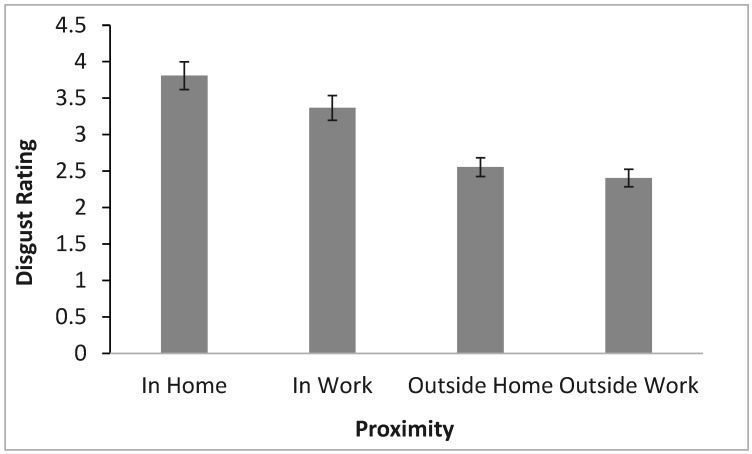
The effect of bed bug proximity on disgust with (mean ± SE).

**Figure 5:**
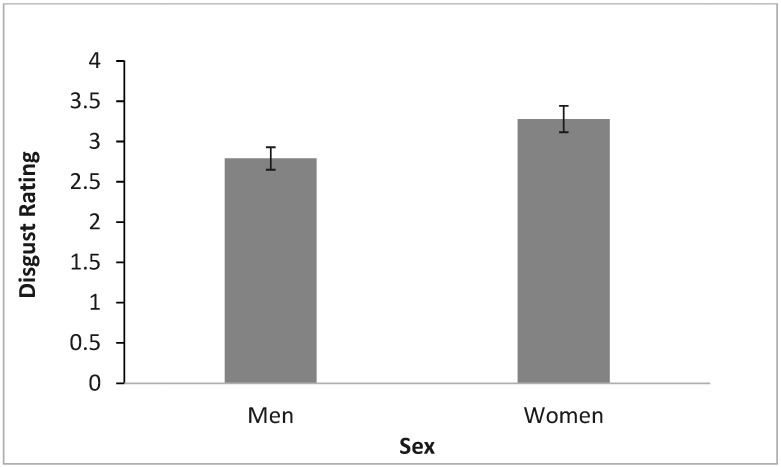
The effect of sex on disgust with (mean ± SE).

**Figure 6:**
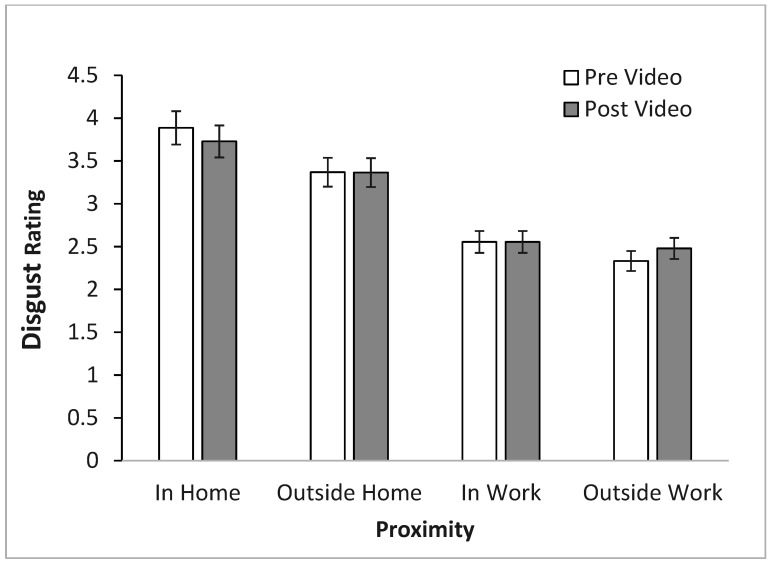
The interaction of time and proximity on disgust with (mean ± SE).

Again, a three-way mixed ANOVA was conducted to determine the effects of time, proximity, and sex on disgust ratings. For disgust, proximity (*F*(3, 307.71) = 239.4, *p* < 0.001) and sex (*F*(1, 143) = 12.85, *p* < 0.001) emerged as significant factors, along with the proximity-time interaction (*F*(2.522, 360.59) = 4.54, *p* = 0.006). Participants reported highest disgust levels for home infestations (Figure 4), and females consistently reported higher disgust than males (Figure 5). The proximity-time interaction showed a decreased disgust for bed bugs in home scenarios post-video, but increased disgust for bed bugs in outside-the-home scenarios (Figure 6).

### 3.3. Educational Video Intervention Enhances Public Knowledge of Bed Bug Management

**Figure 7:**
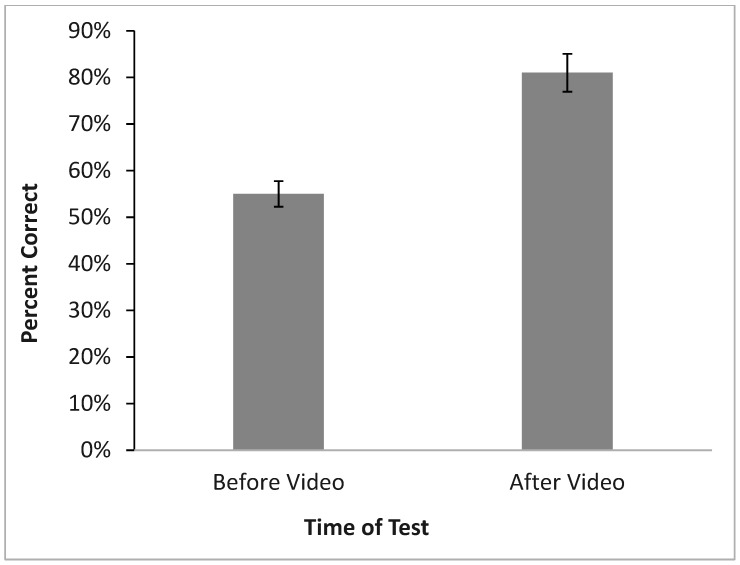
Before and after bed bug knowledge test results with (mean ± SE).

Participants’ knowledge about bed bugs was tested prior to exposure to a short (~10 min), self-administered video recorded by the researchers. Results from the pretest showed that before the study, participants were able to score an average of 9.37 (55%) on the knowledge test, demonstrating, with a failing grade, a deficit in their knowledge about bed bugs. After the study, participants were given the same knowledge test and scored 13.63 (81%) on average, a passing grade. A paired-samples *t* test revealed the knowledge difference was statistically significant, *t*(156) = −27.31, *p* < 0.001 showing the incredible effectiveness of even this short knowledge intervention (Figure 7).

## 4. Discussion

Urban students identified insects as their second most fear-inducing subject, surpassed only by snakes, from among 23 categories of fears and discomforts that included (but was not limited to) getting lost, personal comfort issues (e.g., thirst), strangers/people (e.g., getting hurt, killers, poisonings), sounds, smells, and disease-causing agents [34]. This fundamental fear response forms one of the psychological foundations of the current research. This ingrained aversion to insects represents an evolutionary adaptation that transcends cultures, as documented by Arrindell and colleagues [35] and Davey and colleagues [36] who found consistent insect phobia patterns across 11 different countries. Understanding this baseline emotional response is essential for developing effective interventions that acknowledge rather than dismiss these emotional reactions. As previously discussed, one specific lack in the literature to date has been understanding the emotional perceptions of bed bugs. The present research has helped to close this gap.

The critical significance of this research lies in its potential to transform pest management strategies by understanding emotional drivers that influence resident cooperation and compliance. Additionally, given that pest control professionals typically handle bed bug management rather than residents, these findings could help professionals improve treatment success by addressing clients’ emotional concerns, enhancing communication to reduce fear-driven non-compliance, and using educational strategies that increase residents’ confidence and cooperation throughout the treatment process.

As demonstrated by Etkin and Wager [37], emotional responses often override rational decision-making when confronting threats, leading to counterproductive behaviors (“amygdala hijack”) that can aggravate rather than resolve these threats [38], in this case, insect infestations. By mapping these emotional landscapes, our research provides the missing psychological framework necessary for developing evidence-based interventions that align with, rather than contradict, natural human responses to perceived threats.

Our initial hypothesis suggested that fear and disgust would be the only emotions elicited in response to bed bugs and we chose, *a priori*, to examine how fear and disgust interacted with proximity and sex because they are the two active withdrawal emotions we know from anecdotal evidence that people associate with bed bugs. However, this hypothesis was rejected as individuals also exhibited high levels of anxiety and anger. This unexpected emotional complexity reshapes how we must approach public health interventions. These emotional responses challenge the simplistic fear-based messaging that is typically used by pest management campaigns and aligns with results from Ullah and Hassan [39] that effective public health interventions must address the full spectrum of factors that influence people’s emotions. When dealing with pests, we should create messages that recognize people’s complicated feelings about them. By understanding these emotions rather than ignoring them, we can communicate more effectively about pest problems and solutions.

Following the video, disgust, fear, and anger toward bed bugs increased, while anxiety remained consistently high. Notably, disgust was significantly higher than all other emotions after the test. This is worth noting because disgust plays a crucial role in regulating exposure to harm, making it an effective mechanism for decision-making under risk [40]. It is reasonable to assume that increased knowledge should lead to decreased levels of disgust due to the presence of bed bugs in the home. These reduced feelings of disgust could likely reflect increased self-efficacy. When people gain knowledge about managing a controllable threat in their own environment, disgust (an avoidance emotion) naturally decreases. Curtis and colleagues [41] demonstrated that disgust serves an adaptive function by motivating avoidance of disease threats but diminishes when people feel capable of managing the threat. However, based on our results, increased knowledge gain that led to increased disgust in public settings may represent adaptive vigilance. Research [42,43] has shown that disgust can intensify toward threats that are perceived as less controllable. In the case of bed bugs, since participants cannot control bed bug management in public spaces (e.g., hotels, workplaces, etc.), our educational video content may have heightened their awareness of vulnerability in these settings and appropriately increased their disgust-motivated avoidance.

This pattern suggests that the educational video successfully differentiated between controllable (home) and uncontrollable (public) contexts. Rather than a sample size artifact, this appears to be a sophisticated emotional response where a gain in knowledge (based on our educational video) enhanced both self-efficacy at home and appropriate caution for public settings. This of course is potentially beneficial for both individual well-being in a home environment and preventing bed bug spread in the community.

Pathogen-related and insect-related disgust responses appear to be manifestations of the same psychological construct, and participants perceived insects similarly to how they perceived pathogens [44].

Disgust, fear, and anger are classified as negative, high-arousal emotions. More specifically, disgust and fear are considered withdrawal behaviors, prompting individuals to distance themselves from a negative stimulus, whereas anger is classified as an approach behavior, motivating individuals to act toward a perceived solution. This distinction may help explain common reactions when encountering bed bugs. If individuals primarily experience disgust and fear, they may withdraw from infested belongings or areas, leading to neglectful behaviors such as ignoring the problem in hopes that it resolves on its own. Unfortunately, such avoidance behaviors are likely to contribute to the spread of bed bugs rather than reducing their population. Conversely, individuals who respond with anger may react aggressively in their efforts to eliminate the infestation, sometimes taking irrational or extreme measures that can result in damage to personal or commercial property. In some cases, anger-driven actions may also inadvertently facilitate the spread of bed bugs, as individuals hastily dispose of infested items without considering potential consequences. This emotional distinction represents a real development in understanding why traditional pest control messaging can fail. Wakefield and colleagues [45] showed that high exposure public health campaigns that stimulate negative emotions such as disgust, fear and sadness can increase actions that reduce a threat, but it is also possible that campaigns that trigger withdrawal emotions without providing clear action pathways often result in avoidance behaviors rather than problem resolution. This insight explains the paradoxical finding that increased awareness of bed bug threats sometimes correlates with decreased reporting and treatment rates, as documented by Wang and colleagues [46] in their analysis of urban infestation patterns.

An interesting finding was that anxiety levels remained high and did not significantly change between the pretest and posttest, despite fear increasing. This is particularly noteworthy because anxiety and fear are often considered synonymous. However, anxiety is typically associated with vague, uncertain, or potential threats, whereas fear arises in response to specific, immediate dangers [26]. In this study, participants likely perceived bed bugs as a known threat rather than an ambiguous one, explaining the heightened fear response. Anxiety, on the other hand, is often linked to concerns about contamination or disease and is associated with protective behaviors. Additionally, because anxiety involves behavioral conflict—balancing the need to manage the pest with the urgent desire to eliminate it—it can cause significant stress for affected individuals. The differentiation between anxiety and fear responses to bed bugs aligns with neurobiological research from Davis [47] that shows these emotions activate distinct neural pathways and serve different evolutionary functions. This distinction can also be relevant in the context of pest management because given that fear and anxiety are truly separate entities [48], interventions targeting fear versus anxiety would require fundamentally different approaches to be effective.

Based on the research findings and well-known psychological principles, interventions targeting different emotions in pest management require fundamentally distinct approaches due to the unique behavioral and cognitive patterns associated with each emotion.

When fear is the dominant emotion toward bed bugs, interventions can focus on exposure therapy and systematic desensitization. Cognitive-behavioral therapy research demonstrates that fear responds best to gradual, controlled exposure to the feared stimulus [49]. For bed bug management, this could involve the following: (a) In vivo exposure that starts with images of bed bugs, progressing to videos, then to actual specimens in controlled settings. (b) Imaginal exposure that includes supervised inspection of infested areas with proper protective equipment. (c) Interoceptive exposure that involves helping individuals recognize that physical sensations (itching, crawling feelings) do not necessarily indicate an infestation. The goal of these types of fear-based interventions would be to reduce avoidance behaviors that prevent effective pest management, such as refusing to inspect potential hiding spots or delaying professional treatment due to fear.

When people have anxiety because of bed bugs they can benefit from cognitive restructuring, mindfulness, imaginal exposure, and exposure and ritual therapy [50]. Research shows that generalized anxiety responds better to mindfulness-based approaches and worry-specific interventions [50]. For bed bug anxiety, interventions could include: (a) Cognitive Restructuring which aims to transform biased and unrealistic thoughts about bed bug consequences (for example: “If I get bed bugs, my life will be ruined”), to promote a more adaptive and realistic interpretation of events, (b) Mindfulness Training which reduces a person’s repetitive worry cycles about potential bed bug infestations to discourage negative thinking and distance themselves from negative thoughts, (c) Imaginal Exposure typically requires that an individual write detailed worst-case scenarios of what would happen if they encountered a bed bug or had a bed bug infestation. This type of approach would be used to reduce their emotional impact, and (d) Exposure and Ritual Prevention Therapy exercises would allow for individuals to be exposed to the feared situation (e.g., continuing to live in their home and not throwing out all their belongings given that they suspect the presence of bed bugs) while allowing them to learn to function effectively despite not knowing if bed bugs are present in their immediate surroundings. The goal of anxiety-based interventions in bed bug pest management would be to reduce paralyzing worry and increase tolerance for uncertainty. This would enable individuals to function effectively despite not knowing if bed bugs are present in the area or not. These interventions aim to prevent anxiety-driven avoidance behaviors that can interfere with necessary inspection and treatment activities while promoting rational, evidence-based decision-making rather than excessive or insufficient responses. Such interventions can turn vague, generalized anxiety into specific, actionable awareness that builds confidence in a person’s pest management abilities and reduces secondary problems like sleep disruption and social isolation.

Anger toward bed bugs, while potentially motivating action, can lead to counterproductive behaviors. Anger management in pest contexts could focus on channeling aggressive impulses into constructive pest management behaviors. Behavioral activation (BA) can effectively help people engage with valued activities even when facing unpleasant situations [51], in this case bed bugs. When people encounter aversive situations, BA helps them break avoidance patterns by systematically scheduling and monitoring activities that provide positive reinforcement. In the case of bed bugs, this approach could involve directing anger energy toward a systematic bed bug inspection and treatment protocols. Problem-solving training described by [52] is another anger-based intervention strategy that when used in bed bug pest management, would aim to convert a person’s anger into methodical pest management strategies. Lastly, assertiveness training can effectively help people respond constructively to aversive conditions instead of avoiding them [53]. For the case of dealing with bed bugs, this type of intervention could involve effectively communicating with landlords, neighbors, or pest control professionals about the problem so that it can be remedied.

The results of our research showed that disgust decreased after education, suggesting that information-based interventions can be effective for disgust responses. This aligns with research by Wong and colleagues [54] that shows that disgust responses are primarily cognitive in nature and can be modified by education and targeted behavioral interventions and can change how people think about disgusting situations. This supports the use of cognitive-behavioral approaches for managing disgust-based problems, including those that might arise in pest management contexts like bed bug encounters. Several disgust-based approaches that could be used as pest management for bed bugs include (a) Anti-Disgust Cognitive Intervention and (b) Contamination—Focused Exposure. Salmani and colleagues [55] shows that beliefs about disgust significantly reduced Obsessive–Compulsive Disorder (OCD) severity and disgust propensity while increasing people’s ability to tolerate disgusting situations. In the case of bed bug pest management, an anti-disgust cognitive intervention approach would challenge beliefs about disgust reactions to bed bugs by helping people recognize that disgust, while unpleasant, is not dangerous or harmful. This approach could involve identifying those unpleasant thoughts about bed bug encounters (e.g., “This is unbearable” or “I can’t handle this feeling”) and replacing them with more balanced perspectives (e.g., “This disgust feeling is temporary and manageable”). The goal is to increase acceptance of disgust emotions rather than avoiding bed bug-related situations entirely.

Research by Cougle and colleagues [56] showed that contamination-focused exposure therapy was quite effective for individuals with high disgust propensity and reduced their fear and danger perceptions when encountering spiders. From a bed bug pest management perspective, this could mean systematically exposing individuals to bed bug-related stimuli in a controlled, gradual manner. Like the in vivo exposure in the fear-based interventions, contamination-focused exposure therapy could start with the person looking at pictures of bed bugs, then handling bed bug-related materials (like mattress covers or detection tools), then progressing to inspecting potentially infested areas, and finally conducting thorough bed bug management tasks. This exposure helps reduce both disgust sensitivity and avoidance behaviors that interfere with effective pest control.

Disgust tolerance training is also a part of the contamination-focused exposure therapy and this type of approach, as it pertains to bed bug pest management, could involve teaching specific coping strategies for managing disgust responses during bed bug encounters. This could include mindfulness techniques to observe disgust sensations without judgment, breathing exercises to manage physical disgust reactions, and behavioral strategies to continue pest management activities despite feeling disgusted. The focus would be on building the ability to function effectively in bed bug situations while experiencing disgust rather than eliminating the emotion entirely. These approaches would help people engage more successfully with necessary bed bug prevention and treatment activities by reducing disgust-driven avoidance that often interferes with proper pest management.

Our research proposes that educational interventions successfully shifted responses from contamination-based disgust to threat-specific fear. This transformation is crucial because (a) fear-based responses promote appropriate vigilance and inspection behaviors, (b) reduced disgust decreases avoidance of necessary bed bug pest management activities, and (c) controlled anxiety interventions prevent panic-driven decisions that may worsen infestations. The sex differences found in the study (females showing higher disgust and fear) also suggest that interventions may need sex-specific modifications, with males potentially requiring more education about threat severity and females needing more support for disgust and fear management.

It is possible that individual and contextual factors can create unique emotional profiles that can predict people’s behavioral responses with greater accuracy than demographic factors alone. Therefore, examining the impact of multiple factors such as sex, proximity, and knowledge on emotional responses to bed bugs could reduce the inconsistent outcomes of pest management programs and reveal why previous one-size-fits-all approaches have consistently failed. Investigating these multiple factors that influence emotional responses to bed bug ties into assertions from Lowe and colleagues [57] for adaptive intervention frameworks that respond to individual and situational differences.

Sex differences influenced emotional responses, with females consistently reporting significantly higher levels of disgust and fear toward bed bugs than males, regardless of location (home, workplace, indoor, or outdoor). Several studies also reveal this to be true [58,59,60]. For example, females generally exhibit stronger disgust responses than males due to several biological factors [40]. One key reason is that during pregnancy, inflammatory responses in females are downregulated, leading to an upregulation of disgust-mediated disease avoidance behaviors [61]. Additionally, disgust sensitivity fluctuates in relation to female reproductive physiology [62,63]. These evolutionary adaptations profoundly impact how we must design sex-specific intervention strategies. Research by Tybur and colleagues [64] confirms that disgust sensitivity differences between sexes remain consistent across 30 different nationalities, suggesting a biological rather than cultural basis for these variations. This universal pattern emphasizes the fact that public health messaging should move beyond sex-neutral approaches and acknowledge the significantly different emotional thresholds that influence how receptive men and women are to pest management information and their willingness to engage in preventative behaviors.

Consequently, since disease avoidance requires time, energy, and attention, ancestral women likely developed stronger precautionary behaviors than men due to their maternal and nurturing roles. From an evolutionary perspective, the cost–benefit balance favors the development of sex differences in disgust responses, particularly toward pathogens and pests such as bed bugs [40].

Previous research on sex-related emotional responses by [65] gives an explanation for this difference, the immune response hypothesis, which suggests that women, having stronger immune systems than men, may be more prone to experiencing disgust as a protective mechanism against pathogens [66]. These sex-based emotional differences demand fundamentally different approaches to pest management education.

Given that females showed consistently higher fear and disgust responses than males, educational materials could include female-targeted content emphasizing personal control and self-efficacy. For example, interventions could feature female homeowners successfully managing bed bug infestations. This approach would be similar to those used in health behavior change programs [67] where belief in one’s efficacy to exercise control over a perceived threat can affect the basic processes of personal change that leads to better health and well-being. Male-targeted materials might focus more on technical problem-solving aspects, as males showed lower baseline emotional reactivity. Mastery experiences build confidence in one’s abilities to execute required behaviors [68].

Regarding proximity, participants experienced greater disgust and fear when bed bugs were inside a building compared to outside. Additionally, they were more disgusted by bed bugs in their homes than in their workplaces. Interestingly, before watching the informational video, participants expressed significant disgust toward bed bugs in their homes. However, after viewing the video, they reported reduced disgust toward bed bugs in the home and increased fear of their presence elsewhere. Surprisingly, the proximity-time interaction showed decreased disgust for bed bugs in home scenarios post-video, but increased disgust for bed bugs in outside-the-home scenarios. When people feel differently about pests depending on where they encounter them, this helps us create better public health messages. De Dominicis and colleagues [69] found that location affects how people perceive threats and what they plan to do about them. When pest control messages are adapted to these location-specific emotions, they become more relatable and impactful, and people are much more likely to follow the recommended guidelines. For example, in an area with a history of severe pest outbreaks, the message might emphasize the importance of prevention to avoid future infestations. These results suggest that awareness might reduce home-based disgust responses while heightening vigilance in public spaces, thus potentially limiting the spread of bed bugs.

Given that emotional responses changed due to proximity manipulations, this indicates the need for setting-specific approaches. These approaches could involve workplace training programs that emphasize vigilance and early detection protocols, while residential education could focus on empowerment and management strategies. This dual approach reflects successful context-specific health communication strategies similar to those demonstrated in behavior change interventions involving the Behavior Change Wheel framework that targeted different environmental health threats [70].

Knowledge gained from the video also influenced emotional responses. Education can deeply change how people think and act, which affects many different areas of life. The information provided in the video reduced participants’ disgust, as they became less concerned about contamination or disease transmission. However, their fear increased as they began to perceive bed bugs as a more immediate and specific threat. This aligns with the idea that learning more about an avoided threat allows individuals to better understand their enemy and take proactive steps toward eliminating it. Education does not just give people more information—it can also change how they feel about issues. Goetz and colleagues [71] implied that when education works well, it actually shifts people’s emotional responses, not just what they know. This could explain why some educational programs completely change behavior while others with similar information do not work at all.

Finally, we wanted to determine if a brief, self-directed, educational video could teach participants about bed bugs and their management and control strategies. Our findings confirmed the video did have some effects on emotional perceptions of bed bugs, and it was also effective in increasing knowledge among participants, as indicated by significant improvements in their understanding of the topic. It is possible that the video alleviated some feelings of disgust but the pictures and discussion of bed bugs in the video heightened the saliency effect of fear.

Since knowledge gain modified emotional responses, educational strategies could incorporate metacognitive learning activities that help individuals understand and regulate their emotional responses to different bed bug scenarios. This approach has proven effective in academic settings where students learn to monitor and control their emotional reactions to challenging material [72]. Also, educational materials could include explicit emotional validation (“It’s normal to feel disgusted by bed bugs”) combined with coping strategies that build a sense of competence. This strategy would be similar to successful anxiety management interventions in medical settings [73]. Research shows that interventions that address emotional barriers and self-efficacy beliefs produce higher engagement and willingness to comply [72]. Such interventions could include step-by-step management guides that provide mastery-type experiences that reduce anxiety through structured problem-solving approaches.

## 5. Conclusions

The ultimate implication of this research is to rethink how we communicate about bed bug threats and design interventions that work with, rather than against, our emotional responses to these pests. As demonstrated by Colombo [74] and Kapur [75], interventions that acknowledge and address emotional barriers have higher communication and compliance rates than purely informational approaches. This emotion-centered framework represents a paradigm shift from traditional pest management models focused primarily on technical solutions to a more holistic approach that recognizes human psychology as the critical mediating factor between knowledge and action. If we can design interventions (like the self-guided informational video in this study) that specifically target emotions (unlike the self-guided informational video in this study) to change emotional reactions to bed bugs, we may be able to manipulate the behaviors exhibited in response to bed bugs to align behaviors with pest management best practices. Future studies aim to investigate the rational and irrational behaviors that are born from these emotions.

## Data Availability

Raw data is available from the authors upon request.

## Abbreviations

The following abbreviations are used in this manuscript:

DEQ	Discrete Emotions Questionnaire
ID	Identification Number
QR	Quick Response Code
LSD	Least Significant Difference
BA	Behavioral Activation
OCD	Obsessive–Compulsive Disorder

## Appendix A

Disgust/Fear Emotions Bed Bugs/Butterflies Questionnaire

Use the scale to indicate, “To what extent do you predict you would feel _____:

disgust if you saw a bed bug INSIDE your home:

disgust if you saw a bed bug OUTSIDE your home:

disgust if you saw a bed bug INSIDE your workplace/school:

disgust if you saw a bed bug OUTSIDE your workplace/school:

fear if you saw a bed bug INSIDE your home:

fear if you saw a bed bug OUTSIDE your home:

fear if you saw a bed bug INSIDE your workplace/school:

fear if you saw a bed bug OUTSIDE your workplace/school:

disgust if you saw a butterfly INSIDE your home:

disgust if you saw a butterfly OUTSIDE your home:

disgust if you saw a butterfly INSIDE your workplace/school:

disgust if you saw a butterfly OUTSIDE your workplace/school:

fear if you saw a butterfly INSIDE your home:

fear if you saw a butterfly OUTSIDE your home:

fear if you saw a butterfly INSIDE your workplace/school:

fear if you saw a butterfly OUTSIDE your workplace/school:

## Appendix B

Knowledge Test

Please answer **all** the questions in this section. Select the most appropriate answer based on your knowledge of the bed bug.

1. Bed bugs have:

A. Flattened bodies with leathery wings

B. Yellowish hairs over oval, flattened bodies

C. Jumping legs and shortened wings

D. Oval bodies and eight legs

2. Bed bugs find a host through which cues?

A. Blood from open sores

B. Snores and body movement

C. Carbon dioxide, heat, and body odor

D. Household clutter

3. Bed bugs feed by:

A. Chewing on the skin

B. Piercing/sucking blood with a proboscis

C. Burrowing under the skin

D. Lapping up pools of blood

4. Are bed bugs known to spread disease?

A. Yes

B. No

5. How do bed bugs search for their host?

A. Walking/crawling

B. Flying

C. Jumping

D. Digging

6. Bed bugs are confined to the bedroom.

A. True

B. False

7. When inspecting for bed bugs, you should look for:

A. Live bed bugs

B. Dead bed bugs

C. Bed bug eggs

D. Dark brown/black spots

E. All of these

8. People who think they have been bitten, although they have had no real encounter with a hematophagous (blood sucking) insect may have:

A. Entomophobia

B. Arachnophobia

C. Delusory parasitosis

D. Bugophobia

9. Immature bed bugs look like

A. Caterpillars

B. Small adult bed bugs

C. Worms

D. Ants

10. Bed bug infestations are noted by:

A. Blood or fecal spots and a sweet odor

B. Pepper like droppings

C. Silver scales in cracks and crevices

D. Insects around windows and lights

11. Transporting bed bugs into an area is known as a bed bug?

A. Investigation

B. Inspection

C. Introduction

D. Impact

12. Which tools would be useful for bed bug inspection and monitoring?

A. Climb up traps

B. A magnifying glass

C. A flashlight

D. Tweezers and a container

E. All of these

13. Bed bug infestations are a more common occurrence in the US today for all of the following reasons **except**:

A. Traveling has become more affordable and people are going to more places.

B. People in an effort to save money reuse discarded furniture.

C. People have not seen bed bugs in a long time so they do not recognize the signs.

D. Bed bugs only affect large urban areas.

E. People may not have the money to properly treat a bed bug infestation so the problem goes unresolved.

F. Bed bugs are resistant to many pesticides so are not easily controlled.

14. Bed bug bites:

A. Are painful at the time of the bite.

B. Always present themselves on every person the same way.

C. Frequently appear in rows.

D. Are normally seen only on hands and feet of individuals.

15. Important bed bug prevention techniques include all of these **except**:

A. Avoiding clutter.

B. Not taking home abandoned couches, mattresses, and other furniture.

C. Washing and drying clothing on high heat.

D. Traveling with bed bug prevention repellent.

E. Taking precautions when traveling such as inspecting rooms.

16. The most likely way bed bugs will be transferred from one location to another is:

A. Hitchhiking on clothing and bags

B. Migration

C. Hitchhiking on cats and dogs

D. Flying from one location to another

17. At what temperature should bed bugs be exposed to for at least 1 min before they will die?

A. 200 °F

B. 120 °F

C. 110 °F

D. 85 °F
